# Healthy eating habits and a prudent dietary pattern improve Nanjing international students’ health-related quality of life

**DOI:** 10.3389/fpubh.2023.1211218

**Published:** 2023-11-24

**Authors:** Anita Nyarkoa Walker, Makhala Mary Weeto, Christiana Babymay Priddy, Salimata Yakubu, Margaret Zaitoun, Qianfeng Chen, Bohan Li, Yucong Feng, Yuxia Zhong, Yuandie Zhang, Tao Wei, Solim Essomandan Clémence Bafei, Qing Feng

**Affiliations:** ^1^Department of Nutrition and Food Hygiene, Nanjing Medical University, Nanjing, Jiangsu, China; ^2^Department of Epidemiology and Health Statistics, Nanjing Medical University, Nanjing, Jiangsu, China

**Keywords:** dietary pattern, snacking, wellbeing, disease prevention, vitality, digestive comfort, aesthetic, meal skipping

## Abstract

**Purpose:**

Low-quality dietary practices, such as fast food consumption and skipping meals, deteriorate the quality of life. However, the available studies on diet and health-related quality of life (HRQoL) used matrices not specific to nutrition. Moreover, how diet affects the HRQoL of international students in China is unknown. Therefore, using a cross-sectional study, the effect of dietary patterns and habits on the HRQoL of international students in Nanjing, China, was examined.

**Methods:**

The researchers collected dietary data using a food frequency questionnaire (FFQ) from February to March 2022. Then, the Food Benefit Assessment (FBA) was used to access HRQoL. Finally, the effect of eating habits and dietary patterns on HRQoL was explored using multilinear regression.

**Results:**

Approximately 454 responses were obtained, with the responses mostly from male subjects (56.4%) and those aged 26 years and above (75.6%). The quality of life according to the food consumed was about average for all the constructs except for aesthetics and disease prevention, as 65.8% skipped meals, particularly breakfast (47.8%). Furthermore, three dietary patterns were identified: prudent, Western, and animal protein patterns. Consequently, by skipping breakfast, vitality (β = −2.362, *p* = 0.04), wellbeing (β = −3.592, *p* = 0.007), digestive comfort (β = −4.734, *p* = 0.008), and disease prevention (β = −5.071, *p* = 0.031) were all reduced. However, consuming at least three meals daily enhanced vitality (β = 2.254, *p* = 0.003) and disease prevention (β = 4.441, *p* = 0.019). Furthermore, aesthetics (β = 4.456, *p* = 0.05), physical appearance (β = 5.927, *p* = 0.003), and vitality (β = 3.323, *p* = 0.009) were also significantly increased by healthy dietary patterns. However, a more Westernized diet led to frequent snacking (β = −4.631, *p* = 0.032), a decline in wellbeing (β = −5.370, *p* < 0.001), and discomfort with digestion (β = −5.101, *p* = 0.01). Finally, increased frequency of snacking (β = −6.036, *p* = 0.012), a decrease in wellbeing (β = −4.494, *p* = 0.004), digestive comfort (β = −9.940, *p* < 0.001), physical appearance (β = −4.926, *p* = 0.027), and disease prevention (β = −5.835, *p* = 0.043) were all associated with an increase in animal protein patterns.

**Conclusion:**

This research indicates that healthy eating habits and patterns positively impact international students’ HRQoL. Therefore, the appropriate authorities should advise students to consume healthy foods regularly to improve their HRQoL.

## 1 Introduction

People of all ages, especially students, must preserve their health and quality of life through an active, healthy lifestyle, and a balanced diet (1). As a result, the world is working to enhance these factors that influence their health-related quality of life (HRQoL). HRQoL is a broad and multidimensional measure of an individual’s perceived physical and mental health. It often includes self-perceptions of disease symptoms or health conditions, side effects, functional status across various living domains, and life quality and satisfaction (2–4). Self-perceived health-related quality of life indicates mortality (5, 6). In addition to being used among patients with irritable bowel syndrome (IBS), persistent constipation, fecal incontinence, diabetes, and cancer, researchers also use HRQoL in healthy individuals. It accurately predicts morbidity and mortality, unmet needs, and intervention outcomes (7–9). Due to its significance to health, improving HRQoL and identifying factors affecting it are current priorities for public health professionals (7, 8).

People’s eating habits, including the manner of eating, the type of food eaten, and whom they eat with, are shaped by their social contacts and affect their HRQoL (10). Healthy dietary patterns consisting of more whole grains, vegetables, fruits, low- and non-fat dairy, and lean meat lower the risk of obesity, cardiovascular disease, and some malignancies and also improve QoL (11). For instance, the Mediterranean diet, which includes more fruits, vegetables, seafood, whole grains, olive oil, and other foods, is advantageous to health and improves HRQoL (12–14). Conversely, unhealthy dietary patterns, such as those high in fat, sugar, processed foods, and fewer fruits and vegetables, were linked to a decline in HRQoL (13, 15). Aside from dietary patterns, a person’s quality of life is influenced by eating habits such as skipping meals, especially breakfast, eating late, irregular eating times, and overeating. For instance, regularly eating breakfast is linked to lower body mass index (BMI) (16), better levels of wellbeing (17), and higher levels of life quality (18), but missing meals lowers life quality (15).

Although HRQoL evaluation tools are crucial in nutrition research, most measures are not nutrition specific. For instance, the Short Form-36 (SF-36), the Weight Impact on Quality of Life Tool, the Irritable Bowel Syndrome Quality of Life Tool, and the Gastrointestinal Quality of Life Index questionnaire (19, 20) do not measure the direct effect of foods ingested on the quality of life of consumers. Therefore, it is essential to calculate the total impact of diet on HRQoL using a questionnaires specific for nutrition, such as the Food Benefit Assessment Questionnaire (FBA). The FBA questionnaire evaluates how participants who are healthy or overweight perceive the effects of their food intake. It describes how a person’s health, vitality, sleep, and digestion are affected by their food (21).

International students have traveled from their country of origin to a host country for academic purposes. These students might experience a lower quality of life because they face issues such as negotiating the healthcare system, the pressures of learning a new language, and balancing financial concerns, social connectivity, and anxiety due to isolation from family and friends (22), all of which affect their health compared to their counterparts in their host countries. Additionally, compared to the native population, immigrants have a worse rate of HRQoL (23–25) due to prejudice, socioeconomic hardship, unfavorable working or studying conditions, and climate change, all potential causes of these disparities(26, 27). 

The number of immigrant students in China has drastically expanded in recent years (28), suggesting differences in their HRQOL compared to host students. Furthermore, a prior study in Nanjing indicated that most international students consume mainly Western and meat-heavy diets, which increases non-communicable diseases and lowers quality of life. Nevertheless, as far as we are aware, no research has looked into the HRQoL of immigrant students in China. Therefore, what is the current status of their HRQoL, and how do their dietary patterns and habits influence this aspect of their life? It is expected that healthy nutritional habits and/or patterns will have a positive influence on HRQoL. To achieve this, the HRQoL, dietary patterns, and eating habits of international students in Nanjing were assessed. Then, the impact of dietary patterns and eating habits on HRQoL was investigated. The appropriate authorities can use the findings of this study to customize the actions required to raise the standard of living for international students in Nanjing.

## 2 Materials and methods

### 2.1 Study design, population, and sampling

Nanjing, the capital of Jiangsu province of the People’s Republic of China, is the second-largest city in the East of China and hosts the majority of international students in the province. The researchers purposively recruited international students from universities in Nanjing through social media promotion of the study link from February 1 to March 31, 2022. The survey questionnaire was then pretested among 30 international students at Nanjing Medical University. The final version of the survey was hosted on the “Wenjuanxing” online platform,^1^ designed for online questionnaires, voting, and comments. Thereafter, volunteers from the various schools shared the link to the survey with WeChat groups of international students. The survey allowed only one entry per participant with a specific WeChat account. There was no inducement to take part. Confidentiality was guaranteed as there was no collection of information about personal identity. The survey could be completed in 5–10 min. Participants consented to participate in the survey by selecting “agree to participate.” Individuals who chose “disagree” automatically ended the questionnaire. The study protocol was exempted from review by the research and ethics committee of Nanjing Medical University because it did not collect biological samples or obtain confidential information. However, the ethical standards of the Declaration of Helsinki and its later amendments were adhered to.

### 2.2 Assessment of health-related quality of life

A self-administered Food Benefit Assessment Questionnaire (FBA) was used to assess the subjects’ HRQoL (FBA) (21). The questionnaire has 41 items divided into seven categories: vitality (Ten items), digestive comfort (nine items), wellbeing (six items), disease prevention (six items), aesthetics (five items), snacking (two items), and physical appearance three items. A five-point Likert response scale ranging from 1 (never/certainly not) to 5 (always/certainly) was used to examine these domains. Respondents were asked to think about what they had eaten in the previous 2 weeks while answering the questions. The final scores for each dimension were determined as follows.


$$\mathrm{Final}\;\mathrm{score}=\frac{\mathrm{Raw}\;\mathrm{score}-\mathrm{Minimum}\;\mathrm{score}}{\mathrm{Maximum}\;\mathrm{score}-\mathrm{minimum}\;\mathrm{score}}X\;100$$

The raw score of a dimension is the mean of the items in the dimension.

Minimum score = 1 and Maximum score = 5.

The final score is determined based on a linear transformation of the mean score and ranges from 0 to 100.

Higher scores indicate a higher positive impact or satisfaction from the daily diet.

This survey tool has been approved and validated with high internal consistency. Cronbach’s α for the various components are as follows: vitality (0.91), digestive comfort (0.89), disease prevention (0.88), wellbeing (0.87), aesthetics (0.82), physical appearance (0.79), and snacking (0.81) (21). The individual aspects of the various domains of this questionnaire can be found in Supplementary Table 1.

### 2.3 Assessment of dietary patterns using the food frequency questionnaire and principal component analysis

To gather the nutritional intake of international students in Nanjing, a modified version of a semi-quantitative food frequency questionnaire (FFQ) that has been validated and used among university students (28, 29) was used. The FFQ contained 56 food items in this survey. The following intake frequencies were used: >1 time daily, 1 time daily, 3–6 times per week, 1–2 times per week, 1–3 times per month, and never or rarely. The food items in the FFQ were arranged in a Likert format, with the frequency of consumption in the rows, the type of food, and the serving sizes in the columns. To minimize the errors in the diet data, an FFQ specific to and validated among university students was used (29). Furthermore, before data collection, the questionnaire was pretested among 30 international students from various countries to ensure that the food list covered most of the foods consumed by the students. We then converted the absolute consumption amounts into the daily portions consumed. For the dietary pattern analysis, we condensed the original 55 food items in the FFQ into nine food groups (28). The nine food groups were then input into a principal component analysis (PCA), and a varimax (orthogonal) rotation was performed to construct dietary patterns. The screen plot, parallel analysis, and component interpretability were used to determine the number of components to be retained (30). Each dietary pattern was interpreted using food groups with factor loadings ≥0.4.

### 2.4 Statistical analysis

The Statistical Package for Social Sciences (Version 26; SPSS Inc., Chicago, IL, United States) was used for statistical analysis. The description of the data was done using frequencies, percentages, and mean and standard deviations. Factor scores and eating patterns were found using principal component analysis. The multilinear regression model was employed to determine how their dietary patterns and eating habits affect their health-related quality of life, with a statistical significance level at a value of *p*  <0.05. We controlled for confounders such as sex, age, and monthly expenditure.

### 2.5 Resource identification initiative

IBM SPSS Statistics (RRID: SCR_019096).

## 3 Results

### 3.1 General characteristics, eating habits, and HRQoL of the study population

The 454 respondents were primarily males (56.4%), within the age range of 26 years and above (75.6%), postgraduates or above (72.7%), and mostly from Africa (82.2%). The majority skipped meals (64.8%), particularly breakfast (47.8%), making them eat either once or twice a day (56.8%). As a result, according to the FBA, their quality of life was about average for all the constructs except for aesthetics and disease prevention (Table 1).

**Table 1:** Demographic characteristics of eating habits and health-related quality of life (HRQoL) of international university students in Nanjing (*n* = 454).

Variables		*N* (%) /mean (SD)
Sex		
	Male	256 (56.4)
	Female	198 (43.6)
Age		
	18–25	110 (24.2)
	26 and above	344 (75.8)
Education		
	Undergraduate and below	124 (27.3)
	Postgraduate and above	330 (72.7)
Program of study		
	Medical related	157 (34.6)
	Non-medical related	297 (65.4)
Weight status		
	Underweight	22 (4.9)
	Normal	190 (41.6)
	Overweight	156 (34.5)
	Obese	86 (19.0)
Monthly expenditure		
	Less than 1,000 RMB	99 (21.8)
	Between 1,000 and 3,000 RMB	229 (50.4)
	More than 3,000 RMB	126 (27.8)
Continent		
	Asia	72 (15.9)
	Africa	373 (82.2)
	Others	9 (2.0)
Do you skip meals in China?		
	Yes	294 (64.8)
	No	160 (35.2)
What type of meal do you skip?		
	I do not skip a meal	160 (35.2)
	Breakfast	217 (47.8)
	Lunch and supper	33 (7.3)
	Breakfast and lunch	44 (9.7)
How many times do you eat in a day?		
	Once/twice	258 (56.8)
	Thrice/more	196 (43.2)
Health-related quality of life		
	Snacking	51.83 (18.77)
	Vitality	57.65 (11.03)
	Wellbeing	56.34 (12.96)
	Physical appearance	61.71 (17.38)
	Aesthetics	73.40 (19.87)
	Digestive comfort	59.44 (17.50)
	Disease prevention	73.04 (22.50)

### 3.2 Food groups and factor loading for the three dietary patterns

According to Table 2, the principal component analysis identified three dietary patterns, i.e., traditional, Western, and animal protein. The prudent pattern explained 31.175% and was heavily loaded with vegetables, legumes, seeds and nuts, cereals and grains, eggs, and milk and its products. The variance explained by the Western pattern was 18.688%, with the fast food and drink loadings being high. On the other hand, the animal protein pattern explained approximately 17.856% of the variance and consisted of red meat and other animal products. The food groups of each pattern had factor loadings of >0.4.

**Table 2:** Food groups used in the principal component analysis and the factor loadings of each dietary pattern of the 454 international students in Nanjing.

Food groups	Food items	Prudent pattern	Western pattern	Animal protein pattern
Vegetables	Spinach/other leafy vegetables, tomatoes, ginger/garlic, potato, onion, lady finger (okra), broccoli, brinjal, pumpkin, cabbage/cauliflower, chilly, bell pepper, and others	0.825	-	-
Legumes	Red beans, soybeans, and other beans	0.800	-	-
Seeds and nuts	Groundnuts, almonds, cashew nuts, currants/raisins, and others	0.699	-	-
Cereals and grains and their products	Maize, white flour, brown flour, instant noodles, white rice, brown rice, oats, porridge, bread/toast, biscuit/cake, and others	0.696	-	-
Fruits	Bananas, grapes, apples/pear, mango, orange, pineapple, strawberries, and others	0.682	-	-
Drinks	Tea, coffee, carbonated drinks, and others	-	0.845	-
Dairy product	Milk, cheese, milk powder, yogurt, and others	0.417	-	-
Fast foods	Chips, pizzas, burgers, sandwiches, and others	-	0.547	-
Eggs	Eggs	0.438		
Red meat	Mutton, beef, pork, and others	-	-	0.836
Other animal products	Chicken, fish, shrimp, and others	-	-	0.710
Variance explained		31.175%	18.688%	17.856%

^a^Food groups with absolute factor loadings of ≥ 0.400 are included in each dietary pattern. ^b^Kaiser–Meyer–Olkin measure of sampling adequacy = 0.827. ^c^*p* for Bartlett’s test of sphericity < 0.001. ^d^Rotation method: varimax orthogonal rotation.

**Figure 1:**
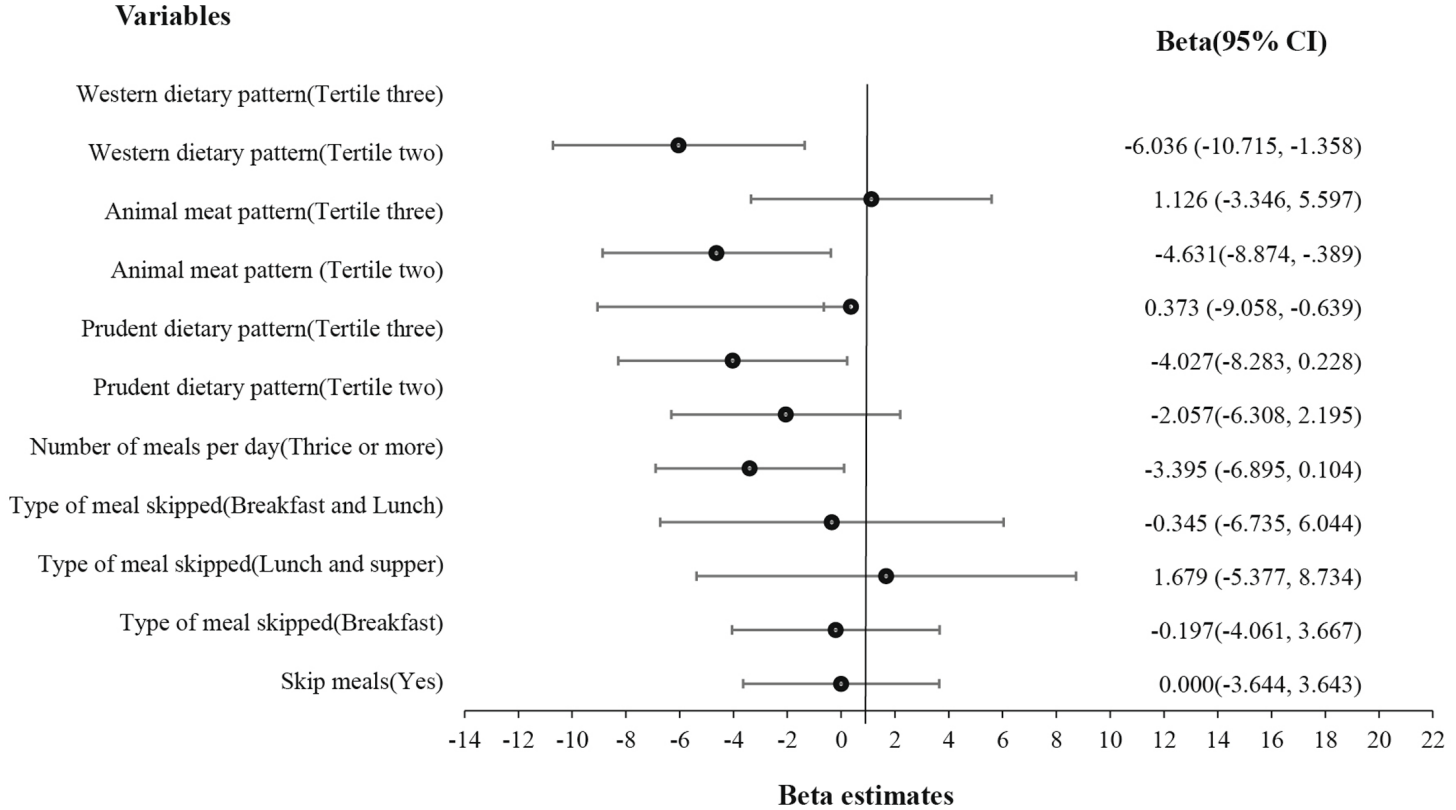
Effect of eating behavior and dietary patterns on snacking.

From Figure 1, the third tertile of all the dietary patterns, Western (β = −6.036, *p* = 0.012), animal meat pattern (β = −4.631, *p* = 0.032), and even prudent dietary pattern (β = −3.395, 0.023) were all associated with an increased snacking behavior.

**Figure 2:**
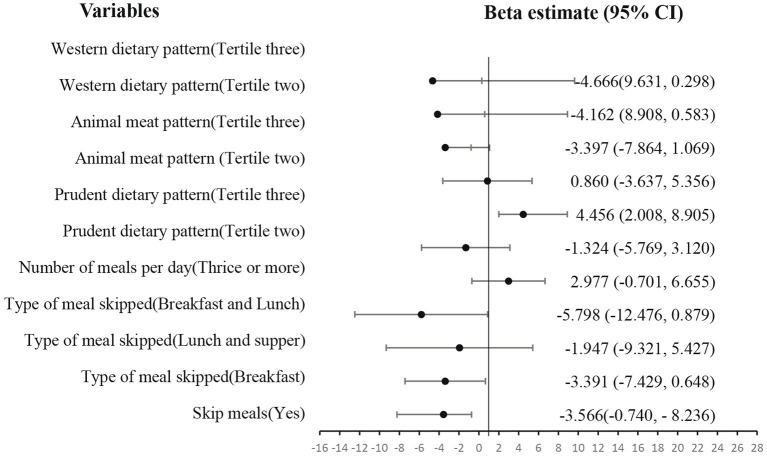
Effect of eating behavior and dietary patterns on aesthetics.

According to Figure 2, skipping meals (β = −3.566, *p* = 0.045) reduced the aesthetics of the students. However, having a prudent dietary pattern (β = 4.456, *p* = 0.05) improved one’s aesthetics.

**Figure 3:**
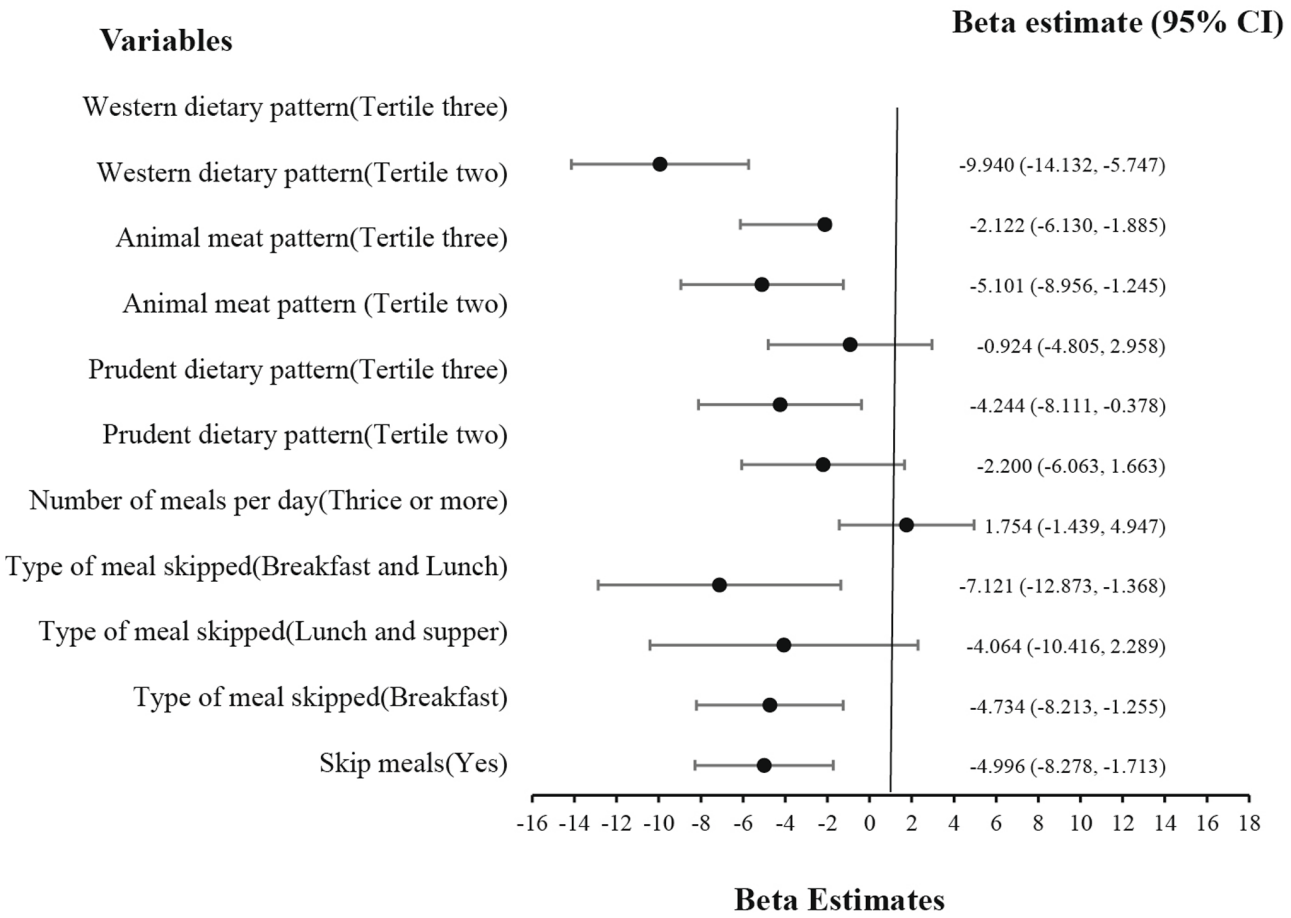
Effect of eating behavior and dietary patterns on digestive comfort.

As shown in Figure 3, digestive comfort was a major problem among our respondents. In this study, skipping meals (β = −4.996, *p* = 0.003), such as breakfast (β = −4.734, *p* = 0.008), or both breakfast and lunch (β = −7.121, *p* = 0.015), all negatively affected their digestive comfort. Similarly, all three dietary patterns, namely, Western (β = −9.940, *p* < 0.001), animal meat pattern (β = −5.101, *p* = 0.01), and prudent dietary pattern (β = −4.244, *p* = 0.032), resulted in lower digestive comfort.

**Figure 4:**
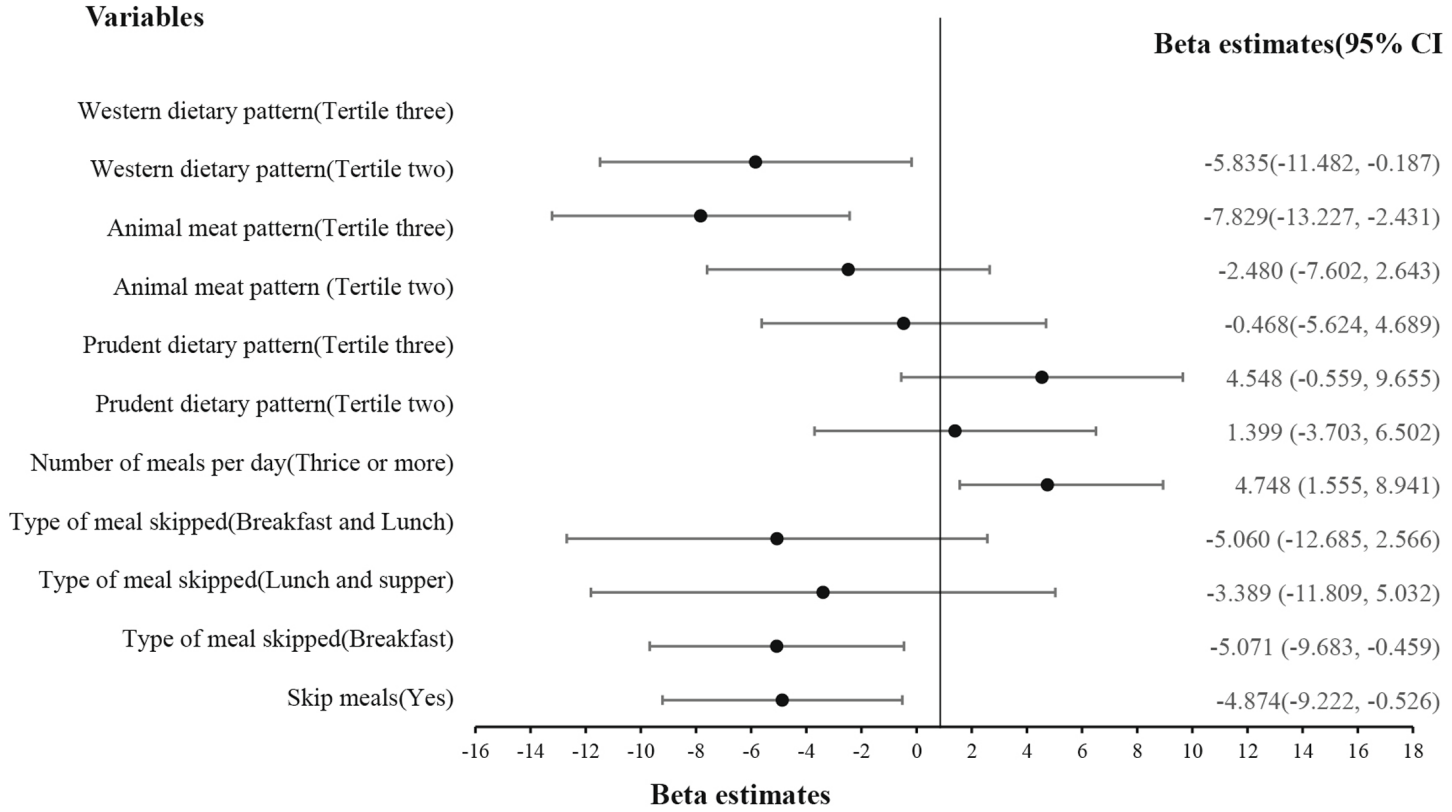
Effect of eating behavior and dietary patterns on disease prevention.

With reference to Figure 4, skipping meals (β = −4.996, *p* = 0.003), especially breakfast (β = −5.071, *p* = 0.031), and consuming a Western dietary pattern (β = −5.835, *p* = 0.043) negatively affected disease prevention, but having a prudent dietary pattern (β = 4.748, *p* = 0.03) increased one’s ability to prevent diseases.

**Figure 5:**
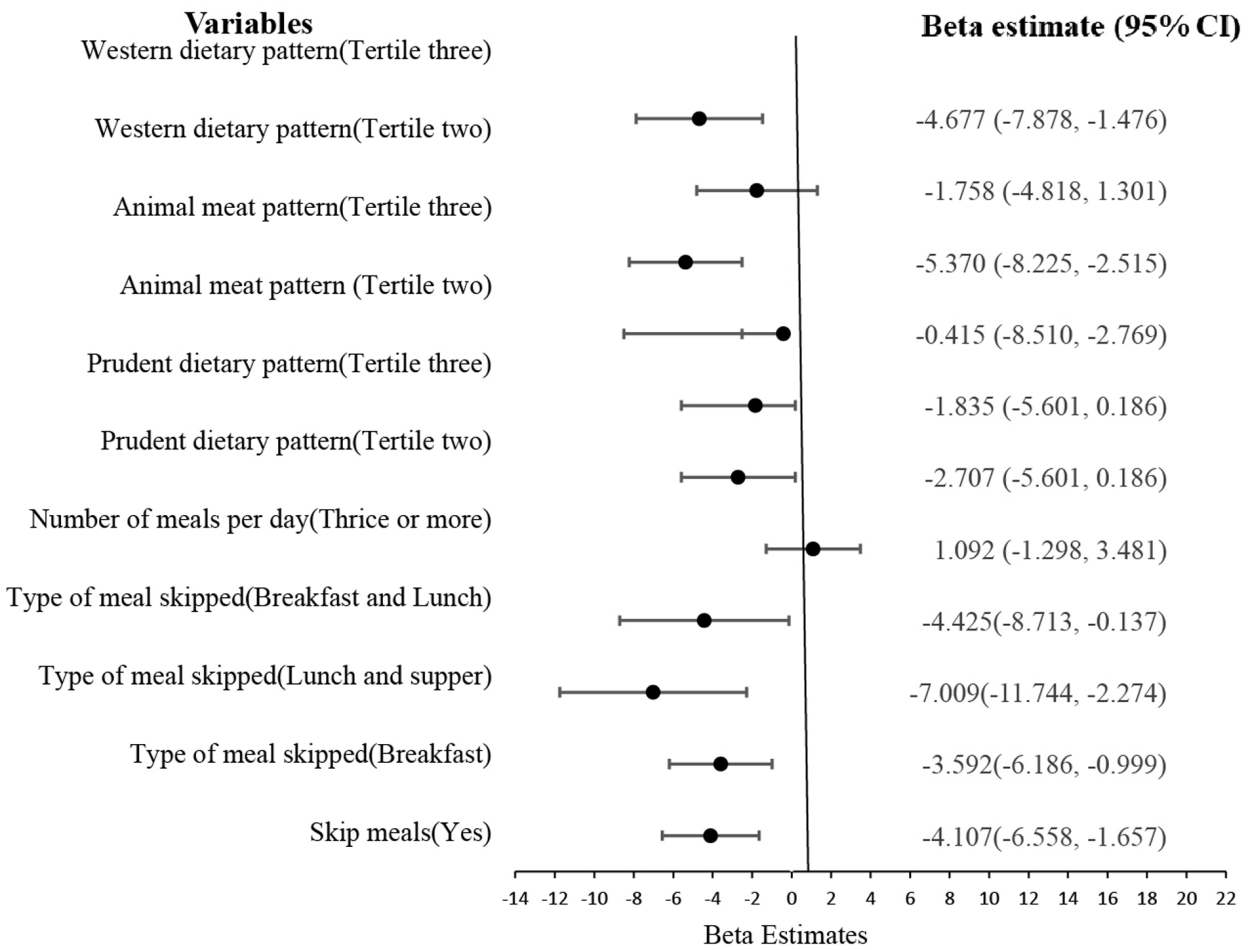
Effect of eating behavior and dietary patterns on wellbeing.

According to Figure 5, skipping meals (β = −4.107, *p* < 0.001) of any type, such as breakfast (β = −5.071, *p* = 0.031), breakfast and lunch (β = −4.425, *p* = 0.043), or even lunch and supper (β = −7.009, *p* = 0.004), reduced wellbeing. In addition, all the dietary patterns such as prudent (β = −1.835, 0.04), animal meat (β = −5.370, *p* < 0.001), and Western (β = −4.494, *p* = 0.004) reduced their wellbeing.

**Figure 6:**
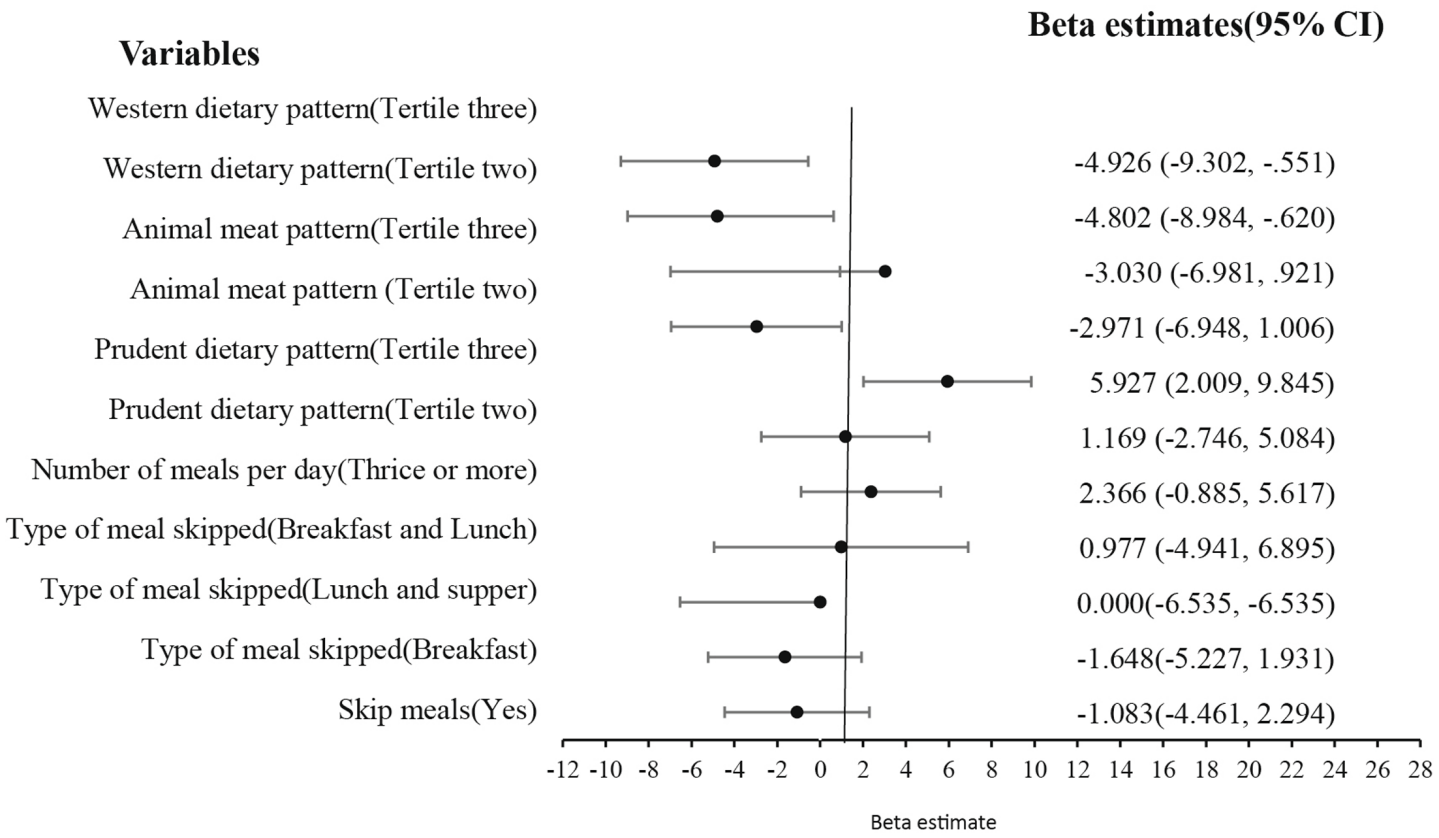
Effect of eating behavior and dietary patterns on physical appearance.

The Western dietary pattern (β = −4.926, *p* = 0.027) reduced while the prudent dietary pattern (β = 5.927, *p* = 0.003) increased the student’s perception of their physical appearance, as shown in Figure 6.

**Figure 7:**
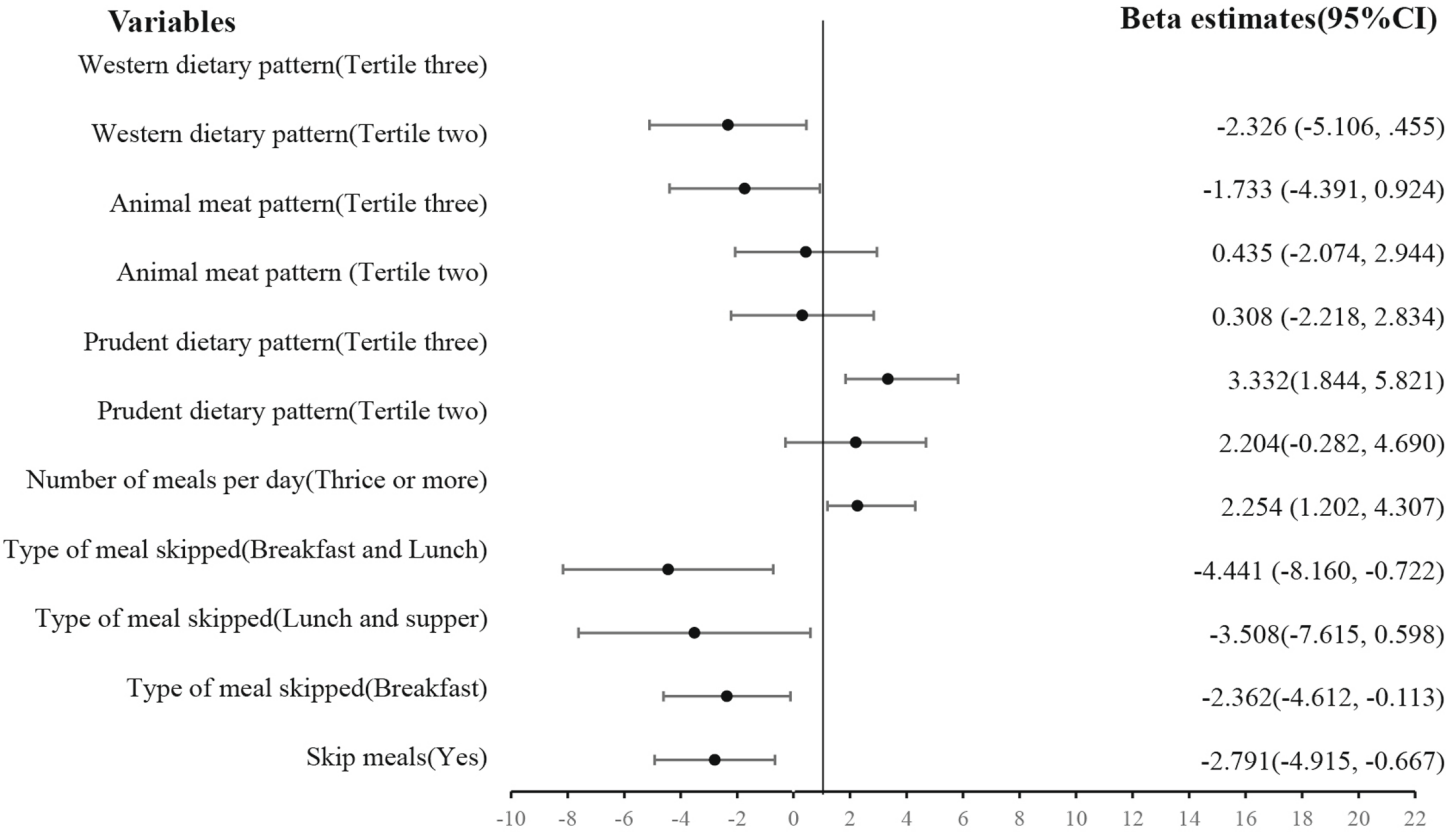
Effect of eating behavior and dietary patterns on vitality.

According to Figure 7, skipping meals (β = −2.791, *p* = 0.01), for example, breakfast (β = −2.362, *p* = 0.04) or both breakfast and lunch (β = −4.441, *p* = 0.019), reduced vitality. However, eating more than thrice a day (β = 2.254, *p* = 0.003) and practicing a prudent dietary pattern (β = 3.323, *p* = 0.009) increased their vitality.

## 4 Discussion

In this cross-sectional study, 454 international students in Nanjing were investigated for their HRQoL, dietary patterns, and eating habits. Research has established that these three variables, namely, sex, age, and income, affect how an individual perceives their quality of life; hence, we controlled them to obtain the actual effect of diet and dietary behavior on their quality of life. For instance, female subjects, young adults, and individuals with high incomes have reported a better quality of life (31–33). This study found that, except for disease prevention and aesthetics, the students’ QoL was about average. Three major dietary patterns were identified: healthy, Western, and animal protein. Finally, unhealthy eating patterns and skipping meals affect HRQoL negatively, while healthy nutritional habits have a positive effect.

Unhealthy eating habits negatively influence vitality, wellbeing, digestive comfort, and disease prevention. Skipping breakfast and lunch was explicitly associated with lower vitality, wellbeing, and digestive comfort. In addition to vitamins A, C, and D, breakfast meals are also good sources of protein, dietary fiber, carbohydrates, fats, and cholesterol (34, 35). Maintenance and improvement of the gastrointestinal system, wellbeing, and vitality require maximum consumption of these nutrients. Hence, meal skippers will suffer poorer wellbeing, vitality, and digestive comfort due to being deficient in these essential nutrients.

Conversely, eating three meals daily can boost vitality and prevent disease. For instance, data from NHANES have shown an increasing meal frequency to reduce cardiovascular mortality (36). Moreover, cross-sectional data have also demonstrated a relationship between lower total and LDL cholesterol levels and more frequent eating (37). This implies that these people will have high vitality and can fend off diseases.

Similarly, healthy dietary patterns have a direct positive relationship with QoL. Accordingly, prudent eating patterns improve vitality, aesthetics, and physical appearance. For instance, similarly to our study, other researchers have established a positive association between vitality and nutritious meals high in natural, plant-based nutrients (38). Furthermore, improved aesthetics and physical appearance require foods rich in healthy nutrients. For example, maintaining good skin, nails, hair, and gums requires meals rich in eggs, seeds and nuts, whole grains, nutritious fats, proteins, dark leafy greens, and fruits (39). This explains why people who follow a healthy eating pattern think highly of their physique and aesthetics features.

Unsurprisingly, poor eating habits lowered QoL. First, a more Westernized diet decreased the wellbeing of the students. Western food consumption is associated with several ailments that affect people’s QoL. Most foods contained in Western dietary patterns are rich in calories from fat, cholesterol, salt, and sugar but low in vitamins, minerals, and other nutrients (40) compared to healthy foods essential for a person’s wellbeing. Second, a high animal protein pattern is associated with poor wellbeing, physical appearance, and disease prevention. Red meat in particular may increase the risk of various forms of long-term diseases when consumed in excess (41, 42). Chronic illnesses reduce how an individual perceives wellbeing and physical appearance. Similarly, those with chronic conditions may believe they have a low capacity for disease prevention.

Furthermore, inappropriate eating habits and skipping meals of the students had a detrimental impact on digestive comfort and snacking. For instance, those who skipped breakfast and those who followed Western and animal protein consumption patterns experienced less digestive comfort. Critical nutrients such as calcium, vitamins, fiber, and others can be found in breakfast foods such as yogurt, milk, and cereal (43). Hence, people who skip breakfast may experience digestive issues because they miss out on the microorganisms and fiber from milk, yogurt, and cereal consumption that aid digestion. Western foods are similarly high in fat, processed sugar, and fiber, leading to the loss of vital microbiomes required for digestion and disease prevention (44). Too much meat consumption also makes it less likely for someone to eat other meals consisting of whole grains, vegetables, and fruits. These may make them feel bloated and occasionally experience constipation due to poor digestion (45).

Moreover, the fatty parts of beef include significant amounts of the sulfur-containing amino acid methionine, which is broken down into offensive gas in the intestines as a result of malabsorption that contributes to indigestion (46). Last but not least, meat typically contains less fiber, an indigestible form of carbohydrate that helps the movement of fecal matter through the stomach and the intestine at a faster pace, aiding digestion and preventing constipation. The factors above could explain why depending on the animal protein pattern severely impacts intestinal comfort. In addition to the digestive function, which meat lacks, fibers also increase satiety by speeding up oral processing and requiring more effort to masticate. These two factors are related to fullness (47). Therefore, people who consume too much animal protein in their diet due to its low fiber content may have low satiety, feel hungry soon after eating, and be at increased risk of snacking between meals.

This study implies that decision-makers can use QoL matrices specific to nutrition to assess how a population’s diet influences their wellbeing. Policymakers and school authorities responsible for international students can encourage international students to regularly consume foods rich in whole grains, nuts, vegetables, fruits, eggs, nuts, legumes, fish, and poultry without skipping meals to improve their quality of life.

The current study’s strength is that it is the first to analyze students’ QoL using the food benefit assessment questionnaire. This questionnaire is specific to the food they consume. Second, the FFQ used is student specific. It does, however, have some limitations. Since the sample size was limited to international students in Nanjing, the findings cannot be extended to include all international students in China. Second, recall bias could arise from the use of the FFQ. Third, comparing our results to previous studies was challenging because this was the first study to use the FBA to determine quality of life. Finally, the cross-sectional design of this investigation precluded the establishment of a causal relationship between the FBA components and dietary patterns and eating behaviors.

### 4.1 Conclusion

In conclusion, this research indicates that healthy eating practices and dietary patterns positively impact international students’ HRQoL. For instance, consuming foods abundant in vegetables, fruits, eggs, nuts, legumes, fish, and poultry improves physical appearance, vitality, and aesthetics. Conversely, Western patterns of drinks, chips, pizza, burgers, and sandwiches, etc., lead to frequent snacking. Additionally, the animal protein patterns of mutton, beef, and pork, etc., are detrimental to wellbeing, physical appearance, and digestive health. Finally, skipping meals such as breakfast increases the problem of digestive discomfort and lowers wellbeing and disease prevention. Nonetheless, eating at least three times every day increases vitality and disease prevention.

## Data availability statement

The original contributions presented in the study are included in the article/Supplementary material, further inquiries can be directed to the corresponding authors.

## Ethics statement

The requirement of ethical approval was waived by Nanjing Medical University, Nanjing, China, for studies involving humans. The studies were conducted in accordance with the local legislation and institutional requirements. The participants provided their written informed consent to participate in this study.

## Author contributions

AW, SY, MW, MZ, QC, BL, YF, YZho, YZha, TW, SB, and QF: study’s conception and design. QF and AW: conceptualization. AW: methodology, software, and writing—original draft preparation. AW and SB: validation. AW and SY: formal analysis. MZ, MW, and SY: investigation. SY: writing—review and editing. QF: supervision. All authors contributed to the article and approved the submitted version.

## Footnotes

^1^ wjx.cn

## References

[1] Marangoni F Martini D Scaglioni S Sculati M Donini LM Leonardi F. Snacking in nutrition and health. Int J Food Sci Nutr. (2019) 70:909–23. doi: 10.1080/09637486.2019.1595543 30969153

[2] Ravens-Sieberer U Erhart M Rajmil L Herdman M Auquier P Bruil J. Reliability, construct and criterion validity of the KIDSCREEN-10 score: a short measure for children and adolescents' wellbeing and health-related quality of life. Qual Life Res. (2010) 19:1487–500. doi: 10.1007/s11136-010-9706-5, PMID: 20668950 PMC2977059

[3] Ravens-Sieberer U Herdman M Devine J Otto C Bullinger M Rose M. The European KIDSCREEN approach to measure quality of life and wellbeing in children: development, current application, and future advances. Qual Life Res Int J Qual Life Asp Treat Care Rehab. (2014) 23:791–803. doi: 10.1007/s11136-013-0428-3, PMID: 23686556 PMC3953538

[4] Revicki DA Kleinman L Cella D. A history of health-related quality of life outcomes in psychiatry. Dialogues Clin Neurosci. (2014) 16:127–35. doi: 10.31887/DCNS.2014.16.2/drevicki, PMID: 25152652 PMC4140507

[5] Netuveli G Pikhart H Bobak M Blane D. Generic quality of life predicts all-cause mortality in the short term: evidence from British household panel survey. J Epidemiol Community Health. (2012) 66:962–6. doi: 10.1136/jech-2011-200310, PMID: 22355079

[6] Otero-Rodríguez A León-Muñoz LM Balboa-Castillo T Banegas JR Rodríguez-Artalejo F Guallar-Castillón P. Change in health-related quality of life as a predictor of mortality in the older adults. Qual Life Res Int J Qual Life Asp Treat Care Rehab. (2010) 19:15–23. doi: 10.1007/s11136-009-9561-4 19946754

[7] Healthy People (2010). Health measure report on health-related quality of life and wellbeing. Office of Disease Prevention and Health Promotion. Available at: https://www.healthypeople.gov/2020/about/foundation-health%02measures/Health-Related-Quality-of-Life-and-Well-Being

[8] Centers for Disease Control and Prevention (2018). Health-related Quality of Life (HRQOL). Available at: https://www.cdc.gov/hrqol/concept.htm

[9] Testa MA Simonson DC. Assessment of quality-of-life outcomes. N Engl J Med. (1996) 334:835–40. doi: 10.1056/NEJM199603283341306 8596551

[10] GBD 2019 Diseases and Injuries Collaborators. Global burden of 369 diseases and injuries in 204 countries and territories, 1990-2019: a systematic analysis for the global burden of disease study 2019. Lancet. (2020) 396:1204–22. doi: 10.1016/S0140-6736(20)30925-9, PMID: 33069326 PMC7567026

[11] Neuhouser ML. The importance of healthy dietary patterns in chronic disease prevention. Nutr Res. (2019) 70:3–6. doi: 10.1016/j.nutres.2018.06.002, PMID: 30077352 PMC6328339

[12] Esteban-Gonzalo L Turner AI Torres SJ Esteban-Cornejo I Castro-Piñero J Delgado-Alfonso Á. Diet quality and wellbeing in children and adolescents: the UP&DOWN longitudinal study. Br J Nutr. (2019) 121:221–31. doi: 10.1017/S0007114518003070 30394237

[13] Hojhabrimanesh A Akhlaghi M Rahmani E Amanat S Atefi M Najafi M. A Western dietary pattern is associated with higher blood pressure in Iranian adolescents. Eur J Nutr. (2017) 56:399–408. doi: 10.1007/s00394-015-1090-z, PMID: 26534856

[14] Muros JJ Salvador Pérez F Zurita Ortega F Gámez Sánchez VM Knox E. The association between healthy lifestyle behaviors and health-related quality of life among adolescents. J Pediatr. (2017) 93:406–12. doi: 10.1016/J.JPED.2016.10.005 28130968

[15] Wu XY Zhuang LH Li W Guo HW Zhang JH Zhao YK. The influence of diet quality and dietary behavior on health-related quality of life in the general population of children and adolescents: a systematic review and meta-analysis. Qual Life Res Int J Qual Life Asp Treat Care Rehab. (2019) 28:1989–2015. doi: 10.1007/s11136-019-02162-4, PMID: 30875010

[16] Moreno LA Rodríguez G. Dietary risk factors for the development of childhood obesity. Curr Opin Clin Nutr Metab Care. (2007) 10:336–41. doi: 10.1097/MCO.0b013e3280a94f59 17414504

[17] Lloyd HM Rogers PJ Hedderley DI Walker AF. Acute effects on mood and cognitive performance of breakfasts differing in fat and carbohydrate content. Appetite. (1996) 27:151–64. doi: 10.1006/appe.1996.0042, PMID: 8937619

[18] Chen X Sekine M Hamanishi S Wang H Gaina A Yamagami T. Lifestyles and health-related quality of life in Japanese school children: a cross-sectional study. Prev Med. (2005) 40:668–78. doi: 10.1016/j.ypmed.2004.09.034, PMID: 15850863

[19] Patrick DL Drossman DA Frederick IO DiCesare J Puder KL. Quality of life in persons with irritable bowel syndrome: development and validation of a new measure. Dig Dis Sci. (1998) 43:400–11. doi: 10.1023/a:1018831127942, PMID: 9512138

[20] Reilly WT Talley NJ Pemberton JH Zinsmeister AR. Validation of a questionnaire to assess fecal incontinence and associated risk factors: fecal incontinence questionnaire. Dis Colon Rectum. (2000) 43:146–53. doi: 10.1007/BF02236971 10696886

[21] Guyonnet D Chassany O Picard C Guillemin I Meunier J Seignobos E. Perceived subject outcomes and impact on health-related quality of life associated with diet using the new food benefits assessment (FBA) questionnaire: development and psychometric validation. Public Health Nutr. (2008) 11:1163–72. doi: 10.1017/S1368980008001729, PMID: 18279564

[22] Vakkai RJY Harris K Crabbe JJ Chaplin KS Reynolds M. Sociocultural factors that impact the health status, quality of life, and academic achievement of international graduate students. J. Int. Stud. (2020) 10:758–75., PMID: 28255647

[23] Brand T Samkange-Zeeb F Ellert U Keil T Krist L Dragano N. Acculturation and health-related quality of life: results from the German National Cohort migrant feasibility study. Int J Public Health. (2017) 62:521–9. doi: 10.1007/s00038-017-0957-6, PMID: 28255647

[24] Sand G Gruber S. Differences in subjective wellbeing between older migrants and natives in Europe. J Immigr Minor Health. (2018) 20:83–90. doi: 10.1007/s10903-016-0537-5, PMID: 27942936 PMC5772126

[25] Toselli S Gualdi-Russo E Marzouk D Sundquist J Sundquist K. Psychosocial health among immigrants in central and southern Europe. Eur J Pub Health. (2014) 24:26–30. doi: 10.1093/eurpub/cku100, PMID: 25107995

[26] Cho S Lee H Oh EG Kim GS Kim Y-C Park C-G. Health-related quality of life among migrant workers: the impact of health-promoting behaviors. Nurs Health Sci. (2020) 22:318–27. doi: 10.1111/nhs.12660, PMID: 31667923

[27] Yoon E Hacker J Hewitt A Abrams M Cleary S. Social connectedness, discrimination, and social status as mediators of acculturation/enculturation and wellbeing. J Couns Psychol. (2012) 59:86–96. doi: 10.1037/a0025366, PMID: 21895356

[28] Haq IU Mariyam Z Zeb F Jiang P Wu X Shah J. Identification of body composition, dietary patterns and its associated factors in medical university students in China. Ecol Food Nutr. (2020) 59:65–78. doi: 10.1080/03670244.2019.1663350, PMID: 31496279

[29] Lupi S Bagordo F Stefanati A Grassi T Piccinni L Bergamini M. Assessment of lifestyle and eating habits among undergraduate students in northern Italy. Annal Dell Istitut Super Sanita. (2015) 51:154–61. doi: 10.4415/ANN_15_02_14, PMID: 26156187

[30] Tabachnick BG Fidell LS In: Row H, editor. Using Multivariate Statistics. 6th ed: Pearson (2013) Allyn & Bacon:Pearson Education

[31] Tesch-Roemer C Motel-Klingebiel A Tomasik M. Gender Differences in Subjective Well-Being: Comparing Societies with Respect to Gender Equality. Social Indicators Research. (2020) 85, 329–349. doi: 10.1007/s11205-007-9133-3

[32] Onadja Y Bignami S Rossier C Zunzunegui M.-V. The components of self-rated health among adults in Ouagadougou, Burkina Fas. Population Health Metrics. (2013) 11:15. doi: 10.1186/1478-7954-11-15 23926951 PMC3750468

[33] Campos ACV e Ferreira EF Vargas AMD Albala C. Aging, Gender and Quality of Life (AGEQOL) study: factors associated with good quality of life in older Brazilian community-dwelling adults. Health and Quality of Life Outcomes. (2014) 12:166. doi: 10.1186/s12955-014-0166-4, PMID: 25433521 PMC4261579

[34] Balvin Frantzen L Treviño RP Echon RM Garcia-Dominic O DiMarco N. Association between frequency of ready-to-eat cereal consumption, nutrient intakes, and body mass index in fourth- to sixth-grade low-income minority children. J Acad Nutr Diet. (2013) 113:511–9. doi: 10.1016/j.jand.2013.01.006, PMID: 23465566

[35] Deshmukh-Taskar PR Nicklas TA O'Neil CE Keast DR Radcliffe JD Cho S. The relationship of breakfast skipping and type of breakfast consumption with nutrient intake and weight status in children and adolescents: the National Health and nutrition examination survey 1999-2006. J Am Diet Assoc. (2010) 110:869–78. doi: 10.1016/j.jada.2010.03.023, PMID: 20497776

[36] Chen H-J Wang Y Cheskin LJ. Relationship between frequency of eating and cardiovascular disease mortality in U.S. adults: the NHANES III follow-up study. Ann Epidemiol. (2016) 26:527–33. doi: 10.1016/j.annepidem.2016.06.006, PMID: 27397905 PMC4993679

[37] St-Onge M-P Ard J Baskin ML Chiuve SE Johnson HM Kris-Etherton P. Meal timing and frequency: implications for cardiovascular disease prevention: a scientific statement from the American Heart Association. Circulation. (2017) 135:e96–e121. doi: 10.1161/CIR.0000000000000476, PMID: 28137935 PMC8532518

[38] Jackson CE DiPlacido J. Vitality as a mediator between diet quality and subjective wellbeing among college students. J Happiness Stud. (2020) 21:1617–39. doi: 10.1007/s10902-019-00150-6

[39] Arakelyan H. (2020). Foods for healthy hair, skin, and nails.

[40] Keshari P Mishra C. Growing menace of fast food consumption in India: time to act. Int J Commun Med Public Health. (2016) 3:1355–62. doi: 10.18203/2394-6040.ijcmph20161600

[41] Forouzanfar MH Lily Alexander H Anderson R Bachman VF Biryukov S Brauer M. Global, regional, and national comparative risk assessment of 79 behavioral, environmental, occupational, and metabolic risks or clusters of risks, 1990-2015: a systematic analysis for the global burden of disease study 2015. Lancet. (2016) 388:1659–724. doi: 10.1016/S0140-6736(16)31679-8, PMID: 27733284 PMC5388856

[42] Zheng Y Li Y Satija A Pan A Sotos-Prieto M Rimm E. Association of changes in red meat consumption with total and cause-specific mortality among US women and men: two prospective cohort studies. BMJ. (2019) 365:l2110. doi: 10.1136/bmj.l2110, PMID: 31189526 PMC6559336

[43] Fanelli S Walls C Taylor C. Skipping breakfast is associated with nutrient gaps and poorer diet quality among adults in the United States. Proc Nutr Soc. (2021) 80:E48. doi: 10.1017/S0029665121000495

[44] Schnorr SL Candela M Rampelli S Centanni M Consolandi C Basaglia G. Gut microbiome of the Hadza hunter-gatherers. Nat Commun. (2014) 5:3654. doi: 10.1038/ncomms4654, PMID: 24736369 PMC3996546

[45] Landverk G. (2020). What eating too much meat can do to your body, from dehydration to the "meat sweats." Insider. Available at: https://www.insider.com/what-eating-too-much-meat-does-health-side-effects-2020-3#your-digestion-might-suffer-from-a-lack-of-fiber-3

[46] Górska-Warsewicz H Laskowski W Kulykovets O Kudlińska-Chylak A Czeczotko M Rejman K. Food products as sources of protein and amino acids-the case of Poland. Nutrients. (2018) 10. doi: 10.3390/nu101219771977, PMID: 30551657 PMC6315330

[47] Miquel-Kergoat S Azais-Braesco V Burton-Freeman B Hetherington MM. Effects of chewing on appetite, food intake, and gut hormones: a systematic review and meta-analysis. Physiol Behav. (2015) 151:88–96. doi: 10.1016/j.physbeh.2015.07.017, PMID: 26188140

