# Supplementary material for: Healthy eating habits and a prudent dietary pattern improve Nanjing international students’ health-related quality of life
Source: Front Public Health. 2023 Nov 24;11:1211218. doi: 10.3389/fpubh.2023.1211218 (PMC10720919; doi:10.3389/fpubh.2023.1211218)
Supplement: Supplementary file 1 [file Table_1.DOCX]

**Dietary behavior, and the health-related quality of life (HRQOL)**

Hello, we are the public health and nutrition research group of Nanjing Medical University, conducting a sample survey among international students in Nanjing. This survey aims to explore the dietary, and the health-related quality of life (HRQOL) of international students in Nanjing. There is no right or wrong answer, and please respond based on your actual situation. Details of respondents will remain anonymous, and the results are solely meant for academic purposes. Hope to get your support.

Do you consent to participate in this survey [单选题] *

| ○Yes |
| --- |
| ○No (结束调查问卷) |

Gender [单选题] *

| ○Male |
| --- |
| ○Female |

Age [单选题] *

| ○18-25 |
| --- |
| ○26-35 |
| ○36-45 |
| ○46 and above |

What is your highest educational level [单选题] *

| ○Undergraduate |
| --- |
| ○Postgraduate |
| ○Doctorate |
| ○Chinese Language Certificate |

What is your Current Program of study [单选题] *

| ○Medical-related Major |
| --- |
| ○non-medical related Major |

What is your Current place of residence [单选题] *

| ○Live in a student dormitory |
| --- |
| ○Live in an apartment in the city or town |

What is your Continent of origin [单选题] *

| ○Europe |
| --- |
| ○Asia |
| ○Africa |
| ○America |
| ○Oceania |

How much is your average monthly living expense? [单选题] *

| ○< 1000 RMB |
| --- |
| ○1000-3000 RMB |
| ○> 3000 RMB |

Do you have any chronic disease [单选题] *

| ○Yes |
| --- |
| ○No |

How many times do you eat in a day [单选题] *

| ○Once |
| --- |
| ○Twice |
| ○Thrice |
| ○More than thrice |

Do you Skip meals frequently in China [单选题] *

| ○Yes |
| --- |
| ○No (请跳至第18题)  What type of meal do you skip? [单选题] *   \| ○Breakfast \| \| --- \| \| ○Lunch \| \| ○Breakfast and Lunch \| \| ○Lunch and Supper \| \|  \| |

What is your current weight [单选题] *

| ○please type here _________________ |
| --- |
| ○I don't know |

What is your current height [单选题] *

| ○please type here _________________ |
| --- |
| ○I don't know |

Please indicate your food intake as best as possible over the past six months[矩阵多选题] *

|  | More than two times/day | 1-2 times/day | 3-6 times/day | 1-3 times/month | Never/seldom |
| --- | --- | --- | --- | --- | --- |
| Tea (1 cup) | □ | □ | □ | □ | □ |
| Coffee (1 cup) | □ | □ | □ | □ | □ |
| Soft drinks (1 cup) | □ | □ | □ | □ | □ |
| Milk(teaspoon) | □ | □ | □ | □ | □ |
| Cheese(teaspoon) | □ | □ | □ | □ | □ |
| Milk powder(teaspoon) | □ | □ | □ | □ | □ |
| Yogurt(one cup) | □ | □ | □ | □ | □ |
| Maize | □ | □ | □ | □ | □ |
| White flour (for Chapati/Naan, Noodles) | □ | □ | □ | □ | □ |
| Brown flour (for Chapati/Naan, Noodles) | □ | □ | □ | □ | □ |
| Instant Noodles | □ | □ | □ | □ | □ |
| White rice (one cup) | □ | □ | □ | □ | □ |
| Brown rice (one cup) | □ | □ | □ | □ | □ |
| Oat (one cup) | □ | □ | □ | □ | □ |
| Porridge | □ | □ | □ | □ | □ |
| Bread/Toast | □ | □ | □ | □ | □ |
| Biscuit/cake | □ | □ | □ | □ | □ |
| Red beans | □ | □ | □ | □ | □ |
| Soya beans (half cup) | □ | □ | □ | □ | □ |
| Eggs (1) | □ | □ | □ | □ | □ |
| Chicken (medium serving) | □ | □ | □ | □ | □ |
| Mutton (medium serving) | □ | □ | □ | □ | □ |
| Beef (medium serving) | □ | □ | □ | □ | □ |
| Pork (medium serving) | □ | □ | □ | □ | □ |
| Fish (medium serving) | □ | □ | □ | □ | □ |
| Shrimps (medium serving) | □ | □ | □ | □ | □ |
| Groundnut (half cup) | □ | □ | □ | □ | □ |
| Almond (half cup) | □ | □ | □ | □ | □ |
| Cashew nuts (half cup) | □ | □ | □ | □ | □ |
| Currants/raisins (medium serving) | □ | □ | □ | □ | □ |
| Carrots (medium serving) | □ | □ | □ | □ | □ |
| Potato (medium serving) | □ | □ | □ | □ | □ |
| Ginger (medium serving) | □ | □ | □ | □ | □ |
| Garlic (medium serving) | □ | □ | □ | □ | □ |
| Tomatoes (medium serving) | □ | □ | □ | □ | □ |
| Spinach and other leafy vegetables (medium serving) | □ | □ | □ | □ | □ |
| Onions (medium serving) | □ | □ | □ | □ | □ |
| Lady finger/ okro (medium serving) | □ | □ | □ | □ | □ |
| Brinjal/ eggplant (medium serving) | □ | □ | □ | □ | □ |
| Pumpkin (medium serving) | □ | □ | □ | □ | □ |
| Cabbage (medium serving) | □ | □ | □ | □ | □ |
| Cauliflow er (medium serving) | □ | □ | □ | □ | □ |
| Chili pepper (medium serving) | □ | □ | □ | □ | □ |
| Bell pepper (medium serving) | □ | □ | □ | □ | □ |
| Banana (medium serving) | □ | □ | □ | □ | □ |
| Grapes (medium serving) | □ | □ | □ | □ | □ |
| Mango (medium serving) | □ | □ | □ | □ | □ |
| Pear (medium serving) | □ | □ | □ | □ | □ |
| Apple (medium serving) | □ | □ | □ | □ | □ |
| Orange (medium serving) | □ | □ | □ | □ | □ |
| Pineapple (medium serving) | □ | □ | □ | □ | □ |
| Strawberries (medium serving) | □ | □ | □ | □ | □ |
| Chips | □ | □ | □ | □ | □ |
| Pizza | □ | □ | □ | □ | □ |
| Burger | □ | □ | □ | □ | □ |
| Sandwich | □ | □ | □ | □ | □ |

Please answer the following questions while thinking about **what you have been eating over the last two weeks**. There are no right or wrong answers; we are just interested in your opinion.[矩阵多选题] *

|  | Always | Often | Sometimes | Rarely | Never |
| --- | --- | --- | --- | --- | --- |
| Have you wanted to eat snacks between meals? | □ | □ | □ | □ | □ |
| Have you felt hungry between regular main meals? | □ | □ | □ | □ | □ |
| Have you felt full of get and go (that is felt great)? | □ | □ | □ | □ | □ |
| Have you been able to carry out all your daily activities efficiently? | □ | □ | □ | □ | □ |
| Have you felt on the ball (attentive and quick to act)? | □ | □ | □ | □ | □ |
| Have you found it easy to concentrate? | □ | □ | □ | □ | □ |
| Have you been dynamic in doing things? | □ | □ | □ | □ | □ |
| Have you felt energetic? | □ | □ | □ | □ | □ |
| Have you felt physically tired? | □ | □ | □ | □ | □ |
| Have you felt suddenly tired before meals? | □ | □ | □ | □ | □ |
| Have you felt mentally tired? | □ | □ | □ | □ | □ |
| Have you felt like dozing after meals? | □ | □ | □ | □ | □ |
| Have you had moments when you felt low? | □ | □ | □ | □ | □ |
| Have you felt stressed? | □ | □ | □ | □ | □ |
| Have you felt on the edge (quickly irritated)? | □ | □ | □ | □ | □ |
| Have you felt calm? | □ | □ | □ | □ | □ |
| Have you felt good psychologically? | □ | □ | □ | □ | □ |
| Have you felt good physically? | □ | □ | □ | □ | □ |
| Have you been happy with your weight? | □ | □ | □ | □ | □ |
| Have your clothes felt tight? | □ | □ | □ | □ | □ |
| Have you looked well? | □ | □ | □ | □ | □ |
| Has your hair looked good? | □ | □ | □ | □ | □ |
| Has your nails looked good? | □ | □ | □ | □ | □ |
| Has your skin looked good? | □ | □ | □ | □ | □ |
| Has your breath smelled okay? | □ | □ | □ | □ | □ |
| Have you had a restless sleep? | □ | □ | □ | □ | □ |
| Have you had a stomach ache? | □ | □ | □ | □ | □ |
| Have you had a heartburn? | □ | □ | □ | □ | □ |
| Have you felt queasy (nauseous)? | □ | □ | □ | □ | □ |
| Have you had a rumbling stomach? | □ | □ | □ | □ | □ |
| Have you felt bloated (stomach feels painfully stuffed, swollen or filled with gas)? | □ | □ | □ | □ | □ |
| Have you burped (belch)? | □ | □ | □ | □ | □ |
| Have you passed wind (fart or flatulate)? | □ | □ | □ | □ | □ |
| Have you had indigestion? | □ | □ | □ | □ | □ |
| Has your stomach felt heavy (sleepiness or heaviness) after eating? | □ | □ | □ | □ | □ |

Please answer the following questions while thinking about **what you have been eating over the last two weeks.** There are no right or wrong answers; we are just interested in your opinion.[矩阵多选题] *

|  | Certainly | Probably | May be | Probably not | Certainly Not |
| --- | --- | --- | --- | --- | --- |
| Can your eating habit help you improve your health? | □ | □ | □ | □ | □ |
| Can your eating habit help you get into better shape physically? | □ | □ | □ | □ | □ |
| Can your eating habit help prevent certain diseases? | □ | □ | □ | □ | □ |
| Can your eating habit help you live longer? | □ | □ | □ | □ | □ |
| Can your eating habit help you appear younger than people of your age? | □ | □ | □ | □ | □ |
| Can your eating habit help you age more healthily? | □ | □ | □ | □ | □ |

Thank you for participating in this survey.
